# Evaluation of Women's Empowerment in a Community-Based Human Papillomavirus Self-Sampling Social Entrepreneurship Program (Hope Project) in Peru: A Mixed-Method Study

**DOI:** 10.3389/fpubh.2022.858552

**Published:** 2022-06-13

**Authors:** Michelle B. Shin, Patricia J. Garcia, Mary Elizabeth Dotson, María Valderrama, Marina Chiappe, Nimmi Ramanujam, Marlee Krieger, Kristjana Ásbjörnsdóttir, Ruanne V. Barnabas, Sarah J. Iribarren, Sarah Gimbel

**Affiliations:** ^1^School of Nursing, University of Washington, Seattle, WA, United States; ^2^School of Public Health, Cayetano Heredia University, Lima, Peru; ^3^Department of Global Health, University of Washington, Seattle, WA, United States; ^4^Department of Biomedical Engineering, Duke University, Durham, NC, United States; ^5^Center for Global Women's Health Technologies, Duke University, Durham, NC, United States; ^6^Duke Global Health Institute, Duke University, Durham, NC, United States; ^7^Calla Health Foundation, Durham, NC, United States; ^8^Department of Epidemiology, University of Washington, Seattle, WA, United States; ^9^Center of Public Health Sciences, University of Iceland, Reykjavik, Iceland; ^10^Division of Infectious Diseases, Massachusetts General Hospital, Harvard Medical School, Boston, MA, United States; ^11^Department of Biobehavioral Nursing and Health Informatics, University of Washington, Seattle, WA, United States; ^12^Department of Child, Family, and Population Health Nursing, University of Washington, Seattle, WA, United States

**Keywords:** cervical cancer, HPV self-sampling, social entrepreneurship, empowerment, community-based cancer screening, Peru

## Abstract

**Introduction:**

Understanding community women's relational and financial empowerment in social entrepreneurship could be the key to scaling up community-based human papillomavirus (HPV) self-sampling programs in low- and middle-income countries. The Hope Project, social entrepreneurship in Peru, trains women (Hope Ladies) to promote HPV self-sampling among other women in their communities. This study aims to evaluate the Hope Ladies' relational and financial empowerment after participating in the program.

**Materials and Methods:**

We evaluated the Hope Ladies' experiences of empowerment in social entrepreneurship using a parallel convergent mixed methods design. The Hope Ladies participated in semi-structured in-depth interviews (*n* = 20) and an eight-questions five-point Likert scale survey that evaluated their relational (*n* = 19)/financial (*n* = 17) empowerment. The interview and the survey questions were developed using three empowerment frameworks: Kabeer's conceptual framework, International Center for Research on Women's economic empowerment indicators, and the Relational Leadership Theory. Deductive content analysis was used to evaluate the interviews with pre-determined codes and categories of empowerment. Descriptive statistics were used to analyze the survey results. Qualitative and quantitative data were integrated through a cross-case comparison of emergent themes and corresponding survey responses during the results interpretation.

**Results:**

All Hope Ladies reported experiencing increased empowerment in social entrepreneurship. *Interviews:* The women reported challenges and improvement in three categories of empowerment: (1) resources (balancing between household and Hope Lady roles, recognition from the community as a resource, camaraderie with other Hope Ladies); (2) agency (increased knowledge about reproductive health, improved confidence to express themselves, and ability to speak out against male-dominant culture); and (3) achievement (increased economic assets, improved ability to make financial decisions, and widened social network and capital, and technology skills development). *Survey*: All (100%) agreed/totally agreed an increase in social contacts, increased unaccompanied visits to a healthcare provider (86%), improved confidence in discussing reproductive topics (100%), improved ability to make household decisions about money (57% pre-intervention vs. 92% post-intervention).

**Conclusions:**

The Hope Ladies reported improved relational and financial empowerment through participating in community-based social entrepreneurship. Future studies are needed to elucidate the relationship between empowerment and worker retention/performance to inform the scale-up of HPV self-sampling social entrepreneurship programs.

## Introduction

Social entrepreneurship is a highly theorized field of knowledge that has rapidly emerged in recent decades. As such, there has been a proliferation of systematic reviews (1, 2), bibliometric studies (3–5), and other projects (6–8) to explore social entrepreneurship and other efforts to set forth research direction and framework for the future (9–11). Social entrepreneurship could be understood as a phenomenon (12) or organizations (1) that leverage economic activities or innovative business models with the mission of creating or implementing positive social change (13–17) rather than personal or shareholder wealth (18, 19).

Social entrepreneurship activities in low- and middle-income countries have often taken the form of microfinance or microcredit programs designed to advance women's economic development (20). Women working in health-oriented social entrepreneurship programs seek to become financially self-sufficient by promoting health or health products rather than being dependent on or being employed by an organization (21). Women-driven social entrepreneurship programs have been shown to empower the entrepreneurs, not only economically but by widening their social network in their communities, enhancing technical skills with earning potential, and challenging the gender norm and their status in families and society (22). Kabeer explains women's empowerment as a process of changes, “by which those who have been denied the capacity for choice gain this capacity” that entails the inter-related, “indivisible” dimensions of resources (pre-conditions), agency (process), and achievements (outcomes) (23, 24). Kabeer also emphasizes that women are embedded, active members within their society, and hence, their empowerment can create social change in those societies where women lack equal power (24). Some scholars support the claims that the elements of empowerment are inherently and essentially embedded in the for-profit social entrepreneurship models (25–27).

In Peru, cervical cancer is the leading cause of cancer deaths in women aged 15–44 (28). The age-standardized incidence rate in Peru is 23.2 per 100,000 women per year, compared to the world average of 13.1 per 100,000 women (28). Despite the clear need for cervical cancer screening, only 52.4% of the women aged over 30 reported having had a Pap test in the last 2 years, according to the Peruvian Demographic and Family Health Survey from 2015 to 2017 (29). Multiple barriers toward achieving high quality and coverage of cytology (also known as Pap test) programs have been identified in Peru even though it is offered as a free service in the public sector, such as unequal regional concentration of lab facilities and clinics, inconsistency of procedures, distance, fear and shame related to the gynecological examination (30–33). Those screened are often lost to follow-up and/or cannot access the necessary treatment due to prohibitive costs or geography (33). The five-year observed survival rate of cervical cancer in metropolitan Lima is only about 50% (34). HPV self-sampling is an alternative strategy that can overcome barriers to screening because additional providers, facilities, and visits are not required for the initial part of the screening. Using a small cytobrush, women can sample themselves through the vaginal canal in the privacy of their home when it is convenient for them. For this reason, the World Health Organization (WHO) recommends HPV self-sampling as an approach to increase screening uptake for women aged 30–60 years (35).

The Hope Project is a social entrepreneurship program initiated by the Universidad Peruana Cayetano Heredia to promote community-based cervical cancer screening through HPV self-sampling in 2018, following a successful pilot in 2015 (36, 37). It has two components: commercial and social. The commercial component offers HPV self-sampling kits and testing with CareHPV® (Qiagen) online to high- and middle-income women in Peru for a higher price (150 Peruvian Soles [PEN], ~43 US Dollars [2020 USD]) to create a sustainable platform to offer subsidized testing to women with fewer resources. The geographical coverage of the social component of the Hope Project encompasses the socioeconomically disadvantaged peri-urban districts of Ventanilla and Mi Perú, a special city project within Ventanilla called “Pachacutec” in the region of Callao. Many residents of these communities are migrants from different regions of Peru who live in these districts to work in the metropolitan area of Lima-Callao (38). Some parts of Ventanilla, namely Nuevo Pachacutec, were established in early 2000 when the government resettled over 7,000 migrant families living in informal housing from another metropolitan area (39). About 30% of the population in Ventanilla, Mi Perú, and Pachacutec live in poverty, and one hospital and 13 community clinics serve the population of about 500,000 (40). Only 10 colposcopy, one cryotherapy, and one loop electrosurgical excision procedure instruments to examine and treat precancerous lesions were found in the public health clinics in the Callao region in 2017 (41), pointing to potential difficulty accessing follow-up care for women with positive cytology or HPV testing. To bridge this gap, the Hope Project donated one colposcope and cryotherapy instrument to a public health clinic in the catchment area.

**Figure 1:**
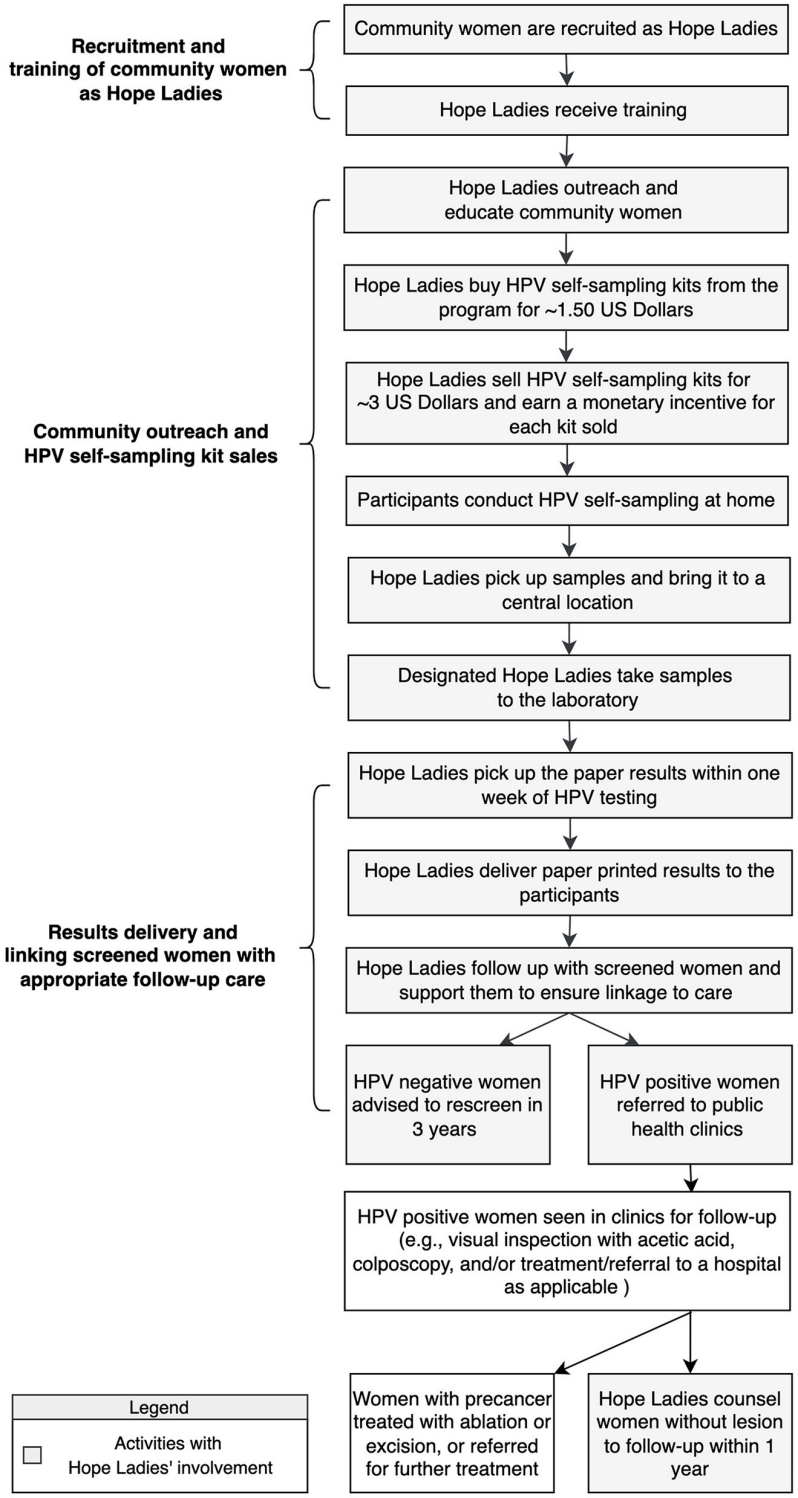
Activities within the Hope Project social entrepreneurship.

In the social component, women from the communities (known as Hope Ladies) are trained to promote cervical cancer screening through HPV self-sampling and guide other women through the screening pathway in their communities. The program activities have been described elsewhere (42) and also described in Figure 1. Briefly, the screening pathway implemented in the Hope Project consists of detecting HPV infection by HPV testing and participants receiving their results via text messages and paper. Women who test negative for HPV are advised to follow up in 3 years, and those who test positive are advised to be evaluated in the public health clinics with visual inspection with acetic acid and colposcopy. HPV positive women with precancerous lesions are treated with cryotherapy if possible or referred to other ablative therapies, and those without lesions are advised to follow up with the public health clinic in 1 year. Women with cancerous lesions are referred for further management in a hospital setting. The Hope Ladies support the participating women through the full screening process by raising community awareness, promoting stigma reduction, distributing kits, and linking women to care with appropriate providers and public health clinics that provide follow-up and treatment as needed.

The concept of women's empowerment has been explored as a mediator between social entrepreneurship and social change (22), but not in the context of HPV self-sampling. Understanding the relationship between these concepts could be the key to informing future program direction and developing scalable social entrepreneurship to increase access to cervical cancer screening. Therefore, we undertook this study to evaluate the Hope Ladies' relational and financial empowerment after participating in the social entrepreneurship. We also developed a causal pathway that can be used to explore empowerment as the mechanism of action and hypothesis generation for the Hope Project intervention in the future.

## Materials and Methods

### Program Setting and Activities

In the social component of the Hope Project, the Hope Ladies initially receive 6 hours of training spanning 2 days on topics including cervical cancer, HPV self-sampling, and project procedures. In addition, the Hope Ladies receive training on effective communication skills, technical skills (e.g., using and managing Whatsapp to communicate with program administrators), and financial skills (e.g., opening and managing online banking, and taxes). Prior to the pandemic, there were monthly monitoring meetings where the Hope Ladies shared their experiences and collectively addressed any challenges from the field.

**Figure 2:**
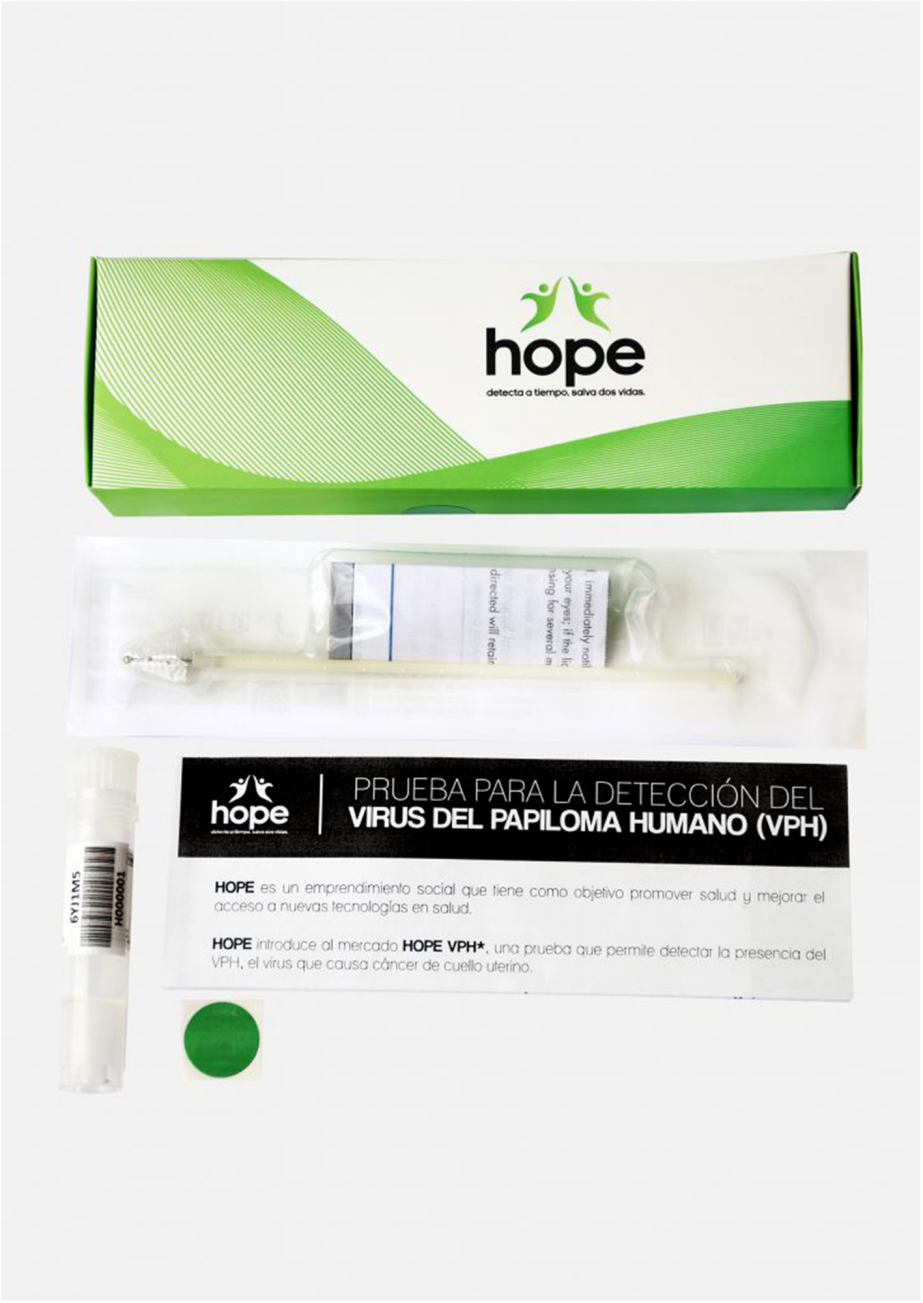
HPV self-sampling kits sold by the Hope Project.

Upon completion of the training, the Hope Ladies buy the HPV self-sampling kits for five PEN per kit (~1.50 USD) from the Hope Project and sell the kits door-to-door by leveraging their social networks in their spare time and keep the small profit of the sales (five PEN per kit [~1.50 USD]) as an incentive for the wide dissemination of the HPV self-sampling kits to their clients. Each kit contains a cytobrush, a collection vial, and a simple instruction that explains how to insert the brush into the vagina and rotate it 3–5 times before placing it into the collection vial (Figure 2). The Hope Ladies educate community women about cervical cancer prevention and how to perform HPV self-sampling. The Hope Ladies pick up the samples from the participants and bring them to a designated central location in the neighborhood, which are then transferred to the laboratory once a week. The Hope Ladies deliver printed test results to the screened women and encourage women who screened positive to follow up in the public health clinics to seek evaluation and treatment as needed. As of March 2022, 62 Hope Ladies had been trained since 2018 and 18 (30%) remain active in the program, although the community outreach has been suspended since March 2020 when the country went into lockdown due to the COVID-19 pandemic.

### Conceptual Framework for Evaluation of Empowerment

We applied three conceptual frameworks to inform the evaluation of empowerment: (1) Kabeer's conceptual model on empowerment (23); (2) International Center for Research on Women (ICRW)'s indicators on women's economic empowerment (43); and (3) relational leadership theory (RLT) (44). We decided to use three frameworks for the analysis because the science of defining and measuring women's empowerment is complex and evolving (45–48), and Kabeer's framework has been successfully used to analyze women's empowerment in social entrepreneurship in developing countries (24, 25).

**Figure 3:**
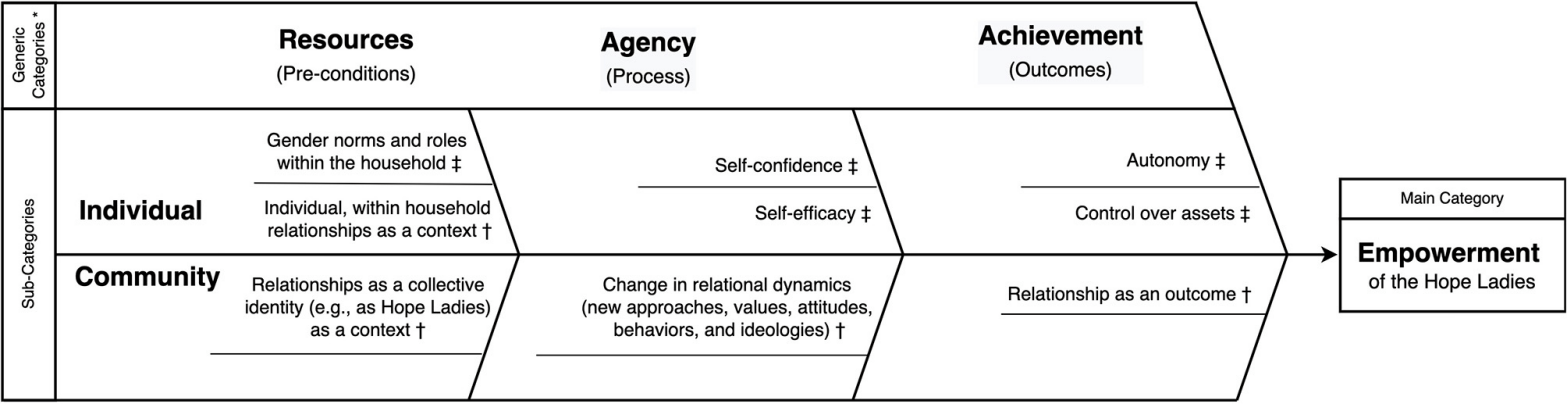
Conceptual framework of financial and relational empowerment. *, Constructs from Kabeer's conceptual framework of empowerment. †, Pre-determined codes about the Hope Ladies' relational empowerment, derived from Relational Leadership Theory. ‡, Pre-determined codes about the Hope Ladies' economic empowerment, derived from International Center for Research on Women.

The relationship between the three conceptual frameworks is depicted in the fishbone matrix diagram in Figure 3. The ICRW's framework measures women's economic empowerment by tracking indicators such as control over assets, agency in decision making, autonomy and mobility, self-confidence and self-efficacy, gender norms, and gender roles within the household (43). Although the measurement indicators have not been specifically validated in Peru among women working in healthcare, we chose this ICRW's framework because it was derived from the literature and the field experience in evaluating women's economic empowerment programs in various low-resource settings. The RLT was used to examine empowerment in the context of family, social networks, and communities, with the assumption that power is “developed and exercised through relationships” (49). The constructs related to financial and empowerment from ICRW framework and RLT were organized into individual- and community-level sub-categories, then mapped onto the generic categories of resource, agency, and achievements from Kabeer's conceptual model of empowerment, acknowledging that these constructs are not mutually exclusive but rather inter-related (23).

### Data Collection and Analysis

We evaluated the Hope Ladies' experiences of empowerment using a parallel convergent mixed methods design (50). To recruit the participants, we selected (via spreadsheet randomization [Microsoft Excel]) and invited 20 of the 62 Hope Ladies (32%) to participate in an individual in-depth interview and a survey through Whatsapp in early March 2020. Each participant gave written informed consent in Spanish. Participant characteristics were gathered from the administrative data from the project. The study was approved by the International Review Boards of Cayetano Heredia University (#103900), Duke University (#2020-0376), and the University of Washington (STUDY00010676).

The interview guide and the eight-question five-point Likert scale-based survey were derived from the ICRW framework and RLT (available as Supplementary Data Sheet). The interview and the survey were designed to take 30 and 10 minutes, respectively, and asked about the Hope Ladies' perceived financial and relational empowerment before and after participating in social entrepreneurship. The interview guide and the survey instruments were prepared in English and translated and reviewed, and piloted by the Hope Project administrators, and amended according to their feedback before finalization.

We conducted individual in-depth interviews and the survey orally in-person in the participants' homes in March 2020. Other family members or program administrators were not present during the interview and the oral survey to minimize bias. Due to the unforeseen logistical challenges and sudden country lockdown due to the COVID-19 pandemic during the week of the data collection, some participants were rescheduled and completed the oral surveys at a later date in June 2021 virtually. The data was collected by at least two trained study personnel, one of whom was fluent in Spanish while the other study personnel took notes.

The interview recordings were transcribed verbatim in Spanish by an independent contractor fluent in Spanish and a resident of a nearby city. Following the structured deductive content analysis method (51), the two authors (MS and MD) independently cleaned the data and organized the emerging themes according to the pre-determined codes from the conceptual framework in Figure 3. They iteratively discussed their individual findings from the qualitative data analysis in a series of meetings and discussed discrepancies until reaching consensus. Salient quotes were translated from Spanish to English by MS and verified by other bilingual authors. The number of participants who endorsed the emergent theme and the reference counts were also measured. Qualitative analysis was performed using NVivo 12 (QRS International, Burlington, MA, USA). Basic frequencies and proportions were used to analyze the results of the survey.

Qualitative and quantitative data were integrated through a cross-case comparison of pre-determined codes from the conceptual framework and corresponding survey questions during the results interpretation (Supplementary Data Sheet). The emergent themes and the reference counts were arrayed in the joint display, along with salient quotes from specific participants and their responses for the respective survey question. Then, the analysis team discussed convergence, divergence and expansion between the qualitative and quantitative data to triangulate and interpret their findings (52).

### Development of Potential Causal Pathway

We developed an implementation science-based causal pathway that can be used for hypothesis generation and for exploring the Hope Ladies' empowerment as the mechanism of action in the future. Using the Agile Science-informed method specified by Lewis et al. and the findings of our surveys and interviews, we developed a pathway model for the Hope Project to increase HPV self-sampling kit sales (proximal outcome), thereby increasing cervical cancer screening coverage (distal outcome) (53). We followed the definition of the mechanism of action, which was the “process or event through which the implementation strategy operates to affect desired implementation outcomes” (53). The cognitive moderator was defined as individual-level perception or attitudes that increase or decrease the level of the influence of the Hope Project. In contrast, the organizational moderator was defined as community-level factors such as culture or widely held beliefs. We also defined pre-conditions, or factors necessary for an implementation mechanism to be activated and the proximal outcome to be realized in the Hope Project.

## Results

All 20 randomly invited Hope Ladies agreed to participate in the study, and the interviews were scheduled for early March 2020. Overall, the 20 Hope Ladies participated in in-person individual in-depth interviews (approximately 60 mins each). Due to the COVID-19 pandemic, five and 10 participants completed the relational and financial empowerment surveys virtually in June 2021, respectively. Ultimately, 19 women completed the relational empowerment survey, and 17 women completed the financial empowerment survey, from which the analysis was conducted.

The demographics of the Hope Ladies are described in Table 1. On average, the Hope Ladies' ages were 45 years old (range: 32–64), had lived in the Ventanilla region for 20 years (range 10–37 years), and had been involved with the Hope Project for 9 months. Most (*n* = 11, 55%) were born in the coastal region, had at least two children (*n* = 18, 90%). All had at least secondary school education, which is equivalent of high school in the United States, and five (25%) had a job in the informal sector other than the Hope Project. Six (30%) were “active” in the Hope Project, defined as sending weekly samples to the laboratory prior to the pandemic, although the level of sales varied greatly throughout the school year when the participants were tending to their children (42). On average, the participants had sold 151 HPV self-sampling kits, with a profit of 23.50 USD per month (Range: 7.20 to 72.80 USD) from the time they began working as Hope Ladies and the time of analysis (August 2020).

**Table 1:** Characteristics of the interview participants (*N* = 20).

**Sociodemographic characteristic**	**Number (%)**
Age (years)	
30–44	10 (50)
45–65	10 (50)
Place of birth	
Andean	7 (35)
Coastal	11 (55)
Amazon	2 (10)
Marital status	
Married/living with partner	16 (80)
Separated	2 (10)
Single	2 (10)
Number of children	
0–2	12 (60)
3–5	8 (40)
Age of last child	
0–5	3 (15)
6–16	12 (60)
17+	6 (30)
Education	
Secondary education	16 (80)
Technical institute	4 (20)
Employment status (informal sector)	
Yes	5 (25)
No	15 (75)

In Table 2, we present the results of the relational and financial surveys. The summary of deductive content analysis of the in-depth interviews is available in the Supplementary Data Sheet with salient quotes from the participants. We present the results organized according to the conceptual framework presented in Figure 3. The cross-case comparison of the qualitative and quantitative data that was used to compare and contrast the emergent themes and the survey responses is available in the Supplementary Data Sheet.

**Table 2:** Relational and financial empowerment survey results.

**Question**	**Number of responses**	**Responses**	***N* (%)**
**Relational empowerment**			
RLT 1.a. Would you say that your number of social contacts within and outside the family has increased since the	*N* = 19	Totally disagree	0 (0)
beginning of your Hope lady journey?		Disagree	0 (0)
		Agree	3 (21)
		Totally agree	16 (84)
RLT 1.b. Would you say that you have been able to help other women in moments of need since the beginning of	*N* = 19	Totally disagree	0 (0)
your Hope lady journey?		Disagree	0 (0)
		Agree	5 (36)
		Totally agree	14 (74)
RLT 1.c. Since becoming a Hope Lady, would you say that you have been able to visit the health care provider to	*N* = 19	Totally disagree	0 (0)
meet your personal needs without your family members or friends accompanying you more easily?		Disagree	2 (14)
		Agree	10 (53)
		Totally agree	7 (37)
RLT 1.d. Would you say that you have felt confident because of learning about your reproductive health and how	*N* = 19	Totally disagree	0 (0)
to prevent certain diseases compared to starting your job Hope lady?		Disagree	0 (0)
		Agree	8 (42)
		Totally agree	11 (58)
**Financial empowerment**			
ICRW 2.a. Currently, do you decide how to spend money in your household?	*N* = 17	Always	16 (94)
		Sometimes	0 (0)
		Rarely	1 (8)
		Never	0 (0)
ICRW 2.b. In the past before joining the Hope Project, did you decide how to spend money in your household?	*N* = 17	Always	7 (41)
		Sometimes	6 (35)
		Rarely	3 (18)
		Never	1 (6)
ICRW 2.c. Currently, are you allowed to comment on the purchase of large domestic assets in the household?	*N* = 17	Always	14 (82)
		Sometimes	1 (6)
		Rarely	2 (12)
		Never	0 (0)
ICRW 2.d. In the past before joining Hope project, were you allowed to comment on the purchase of large	*N* = 17	Always	9 (53)
domestic assets in the household?		Sometimes	6 (35)
		Rarely	2 (12)
		Never	0 (0)

### Resources

Resources could be defined as those conditions that enhance the ability to exercise choice (23). The Hope Ladies described the challenge of managing their roles within the household and working as a Hope Lady and the benefit of peer support within the Hope Project, as well as being recognized as a resource for women's reproductive health in their communities.

#### Gender Norms and Roles Within the Household

##### Maintaining Roles Within the Household and Working as a Hope Lady

The majority of the Hope Ladies who were interviewed (*n* = 15, 75%) mentioned that it is difficult to manage their time to sell HPV self-sampling kits in their communities due to their various roles in their households, such as childrearing and caregiving. One study participant stated, “*I have a baby. When she grows a little more, I don't think I will have any obstacles with the Hope Project”* (Hope Lady, age 35), and another stated, “*I have my mother-in-law in my care. She needs me to take care of her [...] because she cannot get out of bed. I go [out to sell the kits], but with the thought, ‘what if she suddenly falls out of bed,’ or I do not know she will urinate on herself. Sometimes I wonder, ‘Should I continue [to work as a Hope Lady] or not?’ and sometimes I stop [selling the kits]. But my friends [other Hope Ladies] tell me, ‘Don’t stop, keep going for us.”'* (Hope Lady, age 33). Another study participant stated, “*It's definitely not easy [...] it is a matter of organizing, it is a matter of habit, it is a matter of accustoming the family. It has its consequences, but it is possible to balance.”* (Hope Lady, age 33). In contrast, other study participants found it easy or manageable to organize their time. For example, one study participant mentioned, “*It's not difficult for me [to manage my time] because the issue here is to organize ourselves. If we organize ourselves, everything works out for us.”* (Hope Lady, age 45). Another study participant emphasized the convenience of setting their own schedule saying, “*Without having to have an obligatory schedule, in my free time I can go to work myself.”* (Hope Lady, age 33). There were five salient quotes in this theme, and the survey questions corresponding to this theme were RLT 1.a. and RLT 1.b., which asked if the number of social contacts within and outside the family has increased and whether the participant had been able to help other women in the community as a Hope Lady. While all five participants totally agreed/agreed on both survey questions, only one was actively working as a Hope Lady because they were unable to meaningfully engage in the increased social contacts due to their conflicting household roles. Those who paused their Hope Lady role expressed a sense of guilt because they could not continue helping community women. The qualitative and quantitative data diverged in this theme.

#### Relationships at the Community-Level as the Collective Identity of Hope Ladies

##### Hope Ladies as a Resource for the Communities

In the relational empowerment survey, all participants responded “agree/totally agree” (*n* = 19, 100%) to the question, “would you say that you have been able to help other women in moments of need since the beginning of your Hope Lady journey?” (RLT 1.b., Table 2). Half of the Hope Ladies interviewed (*n* = 10, 50%) reported being recognized for their knowledge about cervical cancer in their communities and said, “*They [the community women] talk to me more because you know in the hospital, they [the doctors] will hardly talk to them like we [Hope Ladies] talk to them.”* (Hope Lady, age 45). Both the interview and the survey data showed that being valued as leaders and resource for the community was important to the Hope Ladies.

##### The Camaraderie With Other Hope Ladies

Nearly half (*n* = 9, 45%) of the Hope Ladies interviewed commented on enjoying the peer support with other Hope Ladies and started collaborating with other colleagues helped sell their kits. One study participant said, “*We would agree with other colleagues [Hope Ladies], and we would go out in a group because it is less tedious [than] when you are alone.”* (Hope Lady, age 64). Another study participant commented they look forward to the growth of the Hope Project, saying, “*we are working with the Cayetano [University], so that [the Hope Project] grows and we can amplify the good work.”* (Hope Lady, age 54).

### Agency

Agency is defined as the capability to define one's goal and act upon it (23). The Hope Ladies reported an increased sense of confidence and efficacy in themselves stemming from increased knowledge about reproductive health and improved communication ability and express themselves. They also discussed changes in behaviors, values, attitudes, and ideologies, such as advocating for their clients (other community women) to make autonomous decisions about HPV self-sampling against male-dominant culture (*machismo*).

#### Individual-Level Self-Efficacy and Self-Confidence

##### Increased Knowledge and Self-Efficacy

All (*n* = 20, 100%) of the Hope Ladies said the increased knowledge and education about cervical cancer helped them to make informed decision-making for themselves, as well as other community women. 53% (*n* = 10) agreed, and 37% (*n* = 7) totally agreed that they have been able to increase unaccompanied visits to a healthcare provider to meet their personal needs since the beginning of your job as a Hope Lady?” (RLT 1.c.) One study participant stated, “*It has empowered me, and I have gained a lot of experience […] It taught me to express myself, to reach the families who are the most in need, and I saw that there is a lot of need in the communities that I have visited, and others thank you and tell you, 'Thanks for coming! Thank you for remembering me!' And all that makes your self-esteem rise, and you have more desire to continue working, for them, for them more than anything.”* (Hope Lady, age 47). The four salient quotes in this theme also discussed that many of their HPV positive clients do not want to go to the public health clinic for further evaluation due to fear of cancer diagnosis, encountering a male provider, and long wait time. The interview and the survey data converged in this theme, which demonstrated that the Hope Ladies felt the increased knowledge about the reproductive health gave them confidence to accompany the HPV positive clients to emotionally support and advocate for them during the clinic visits in addition to increasing unaccompanied visits to the healthcare providers for their personal needs.

##### Improved Self-Confidence and Ability to Communicate and Express Thoughts

All study participants agreed (*n* = 8, 42%) or totally agreed (*n* = 11, 58%) in the relational empowerment survey that they felt more confident than before working as a Hope Lady because they learned about the female reproductive health system (RLT 1.d., Table 2). One study participant mentioned, “*If it weren't for this [the Hope Project], I wouldn't even have taken the test,”* (Hope Lady, age 44). More than half (*n* =12, 60%) of the study participants reported improved communication abilities to express themselves. One reported, “*It has helped me to have more confidence in words, that is, in being able to express myself with confidence what I am talking about*.” (Hope Lady, age 33). Another study participant emphasized the importance of ongoing support and training by the Hope Project to her and said, “*I have lost the shame of communicating with people, because before I was not capable. When I started, I was very shy, but now I have enough skills. I have acquired that with [Hope Project] because of the training that they also give us. They support us in everything that we do, we also consult with them.”* (Hope Lady, age 46). The interview and the survey data converged in this theme, which demonstrated that the improved knowledge about reproductive health enhanced the participants' confidence in their ability to learn and perform their roles as Hope Ladies.

#### Change in Relational Dynamics in the Community

##### Advocating for Women Against Male-Dominant Culture

Almost all (*n* = 18, 90%) of the Hope Ladies who were interviewed mentioned male-dominant culture (*machismo*) in the households as a barrier to selling the HPV self-sampling kits. One reported, “*Many times [the women] say, ‘No, my husband does not want [me] to,’ and they have to talk with the husband [to buy the kit]. That is, more than anything*, machismo.” (Hope Lady, age 50). Another participant cited fear as a barrier to screening saying, “*Some women don't do [the HPV self-sampling] out of fear of [their spouses]. They say, ‘my husband will ask me why I’m taking the test! He might think it's because I doubt [his fidelity].'”* (Hope Lady, age 33). A few (*n* = 3, 15%) Hope Ladies reported clients who buy the HPV self-sampling kits in secret, without informing their spouses.

The Hope Ladies stated they advocate for women to make autonomous decisions about their bodies without obtaining permission from their spouses by educating them about the importance of cervical cancer screening. One stated, “*We help them so that they can become aware that the decision is in themselves, and that we do not depend on anyone. We say, ‘We have come alone, and we are going to leave alone, so each one is the owner of what to do and what decisions to make.’ And that is what I have learned with Hope Project.”* (Hope Lady, age 46). Another study participant said, “*The empowerment that [the Hope Project] brings to us, that other institutions cannot, is women's self-realization, their power to decide themselves, not to ask their partner.”* (Hope Lady, age 48). Although the interviewers did not solicit information about domestic violence, a quarter of all study participants (*n* = 5, 25%) mentioned their clients shared that they sometimes experience it.

### Achievements

Achievement can be seen as the outcome of the resources and agency (23). The Hope Ladies reported increased economic assets and expanded social network since joining the Hope Project.

#### Control Over Assets

##### Increased Economic Assets

All (*n* = 20, 100%) study participants reported that the supplemental income from selling HPV self-sampling kits was economically helpful. One study participant responded, “*Of course, it has helped me a lot […] It helps me for my children's bus fares, which is daily for school.”* (Hope Lady, age 45). Another study participant stated, “*Yes, it helps [financially]. It is a job that helps you financially and that you are also helping other people, other women.”* (Hope Lady, age 51). There were three salient quotes in this theme, and the survey questions corresponding to this theme asked if the participant decides how to spend money in their household currently (ICRW 2.a.) and in the past before joining the Hope Project (ICRW 2.b.). All three answered that they “often” make spending decisions currently, whereas their responses varied from “never” to “sometimes” on how often they made spending decisions before joining the Hope Project. The interview and the survey data converged in this theme in that while the participants said financial gains are not the primary motivation for working as Hope Ladies, they derive a sense of achievement from financially contributing to the household.

#### Financial Autonomy

##### Improved Financial Autonomy

Most study participants of the financial empowerment survey (16 of 17, 94%) reported that currently, they “always” decide on how to spend money in their household (ICRW 2.c., Table 2). In contrast, when asked the same question before starting the Hope Project, 41% (7 of 17) responded “always” (ICRW 2.d.). In the individual interviews, the change in the ability to make financial decisions since working as a Hope Lady was more subtle. One study participant who is a single-parent stated, “*I'm the one who works. I am a mother and father, I have a daughter, and I am the one who says how much money comes into my house and how much I am going to spend. I try to balance what is my priority."* (Hope Lady, age 47). Another study participant stated, “*Although I don't [work], I have always tried to solve all the house expenses. [My husband] is the one who contributes.”* (Hope Lady, age 33). Qualitative and quantitative data diverged in this theme in that little change in perceived financial autonomy was observed in the interviews as it was in the survey results.

#### Relationship as an Outcome

##### Widened Social Network and Gaining Technology Skills

In the relational empowerment survey, all study participants responded either “agree” (*n* = 3, 16%) or “totally agree” *(n* = 16, 84%) that the number of social contacts within and outside the family has increased since working as a Hope Lady (RLT 1.a., Table 2). One study participant stated, “*They [the community women] comment on the program and they look for us, and they call us about this topic [of HPV self-sampling]. They call us, they leave our numbers, and other people who have never met call us, and you get to know more people.”* (Hope Lady, age 45).

The widened social network was often discussed in the context of social media and technology skills development. Many study participants mentioned they had limited experience with social media or touchscreen phones before joining the Hope Project. One study participant stated, “*I didn't know how to use [a touchscreen phone] at all. And when I joined the Hope Project, it was practically indispensable…[the program administrators] themselves have taught me to use it, they have taught me to enter the page, to enter the data […] I have learned everything about technology with the Hope Project, because before I didn't even care to pick up a phone, but now I do.”* (Hope Lady, age 46). Another study participant stated, “*Social networks...the cell phone for me was nothing more like the phone that you go and answer, nothing at all! Now I know, well, I chat everything*.” (Hope Lady, age 33). The interview and the survey responses converged in that the combination of technological skills and the widened social network increased the Hope Ladies' sense of achievement.

### Empowerment as the Mechanism of Action

**Figure 4:**
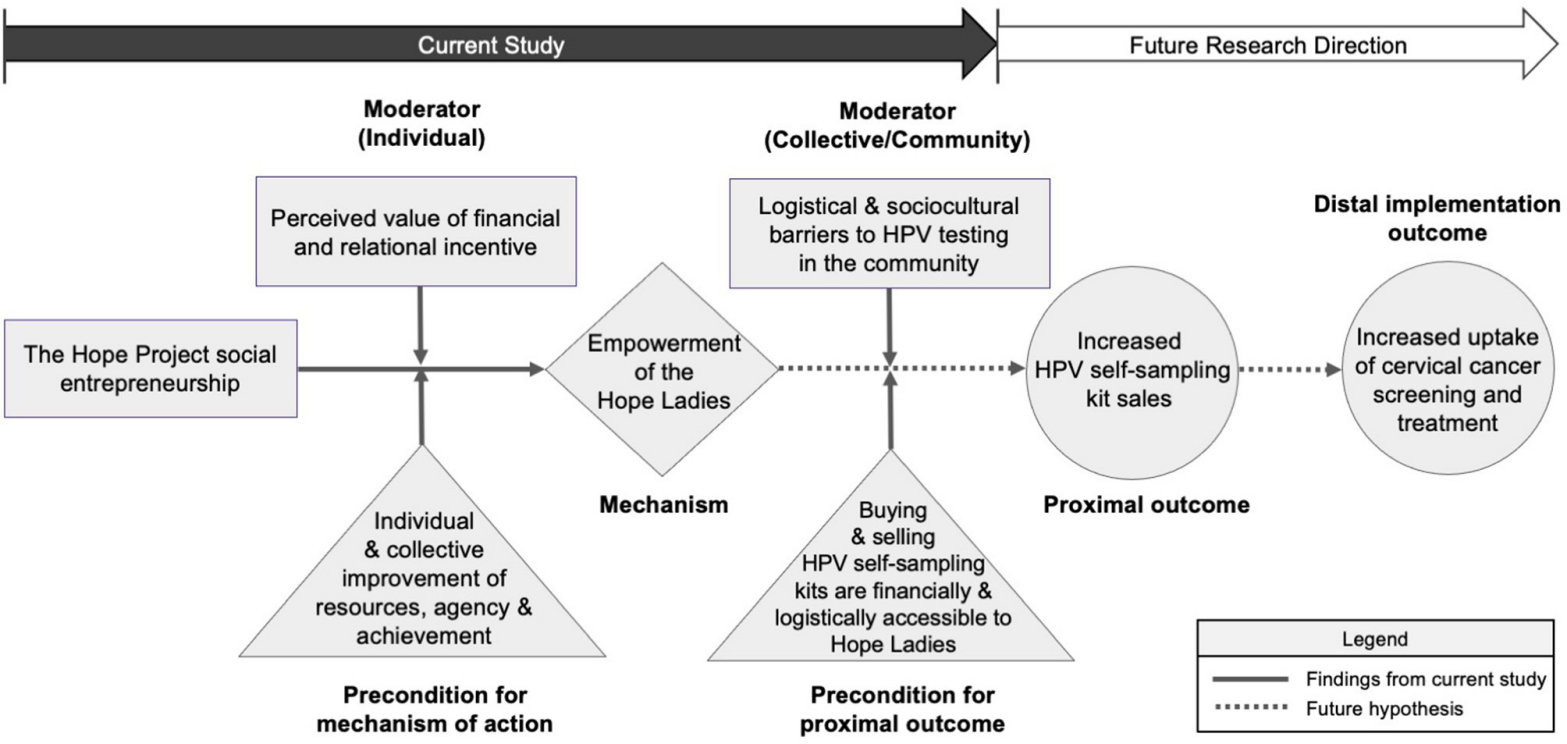
Empowerment as the potential mechanism of action for the Hope Project.

We developed a causal pathway model for the Hope Project based on the mixed methods findings (Figure 4) that can be used for hypothesis generation and testing for the future. We designated the individual and collective improvement of resources, agency, and achievement as the pre-condition for the mechanism of empowerment to be activated because the Hope Ladies discussed how their collective identity of being a resource for their communities, improved self-confidence/efficacy through training and newly gained skills, and financial autonomy helped them to feel empowered in their work as Hope Ladies. We designated the perceived value of financial and relational incentives as cognitive moderators, as they “interact” with the mechanism of empowerment (e.g., widened social network leading to feeling more empowered). Logistical and sociocultural barriers to HPV testing were designated as organizational moderators because the participants discussed how they impacted the numbers of kits sold (e.g., the male-dominant culture in the community interfering with women's ability to buy the kits). The study participants discussed how financial and logistical accessibility was necessary to buy and sell the kits, therefore, we designated it as the pre-condition. We posit that the implementation strategy of microfinancing, training and peer-education operates through the process of Hope Ladies' empowerment to achieve increasing HPV self-sampling kit sales and cervical cancer screening in the Hope Project (proximal and distal outcomes, respectively), which can be tested in future research studies.

## Discussion

We evaluated the relational and financial empowerment of women participating in social entrepreneurship called the Hope Project in Peru using surveys and in-depth interviews and created a pathway model to inform future program direction and scaling of this community-based HPV self-sampling intervention. We found that the Hope Ladies individually and collectively experienced meaningful improvement of resources, agency, and achievement in varying degrees and forms, which expanded their capacity to make strategic and meaningful choices in their households and communities. Using a pathway model, we show how HPV self-sampling kit sales could be achieved through the empowerment of the Hope Ladies, which would function as the mechanism of action for the Hope Project.

There is strong evidence linking women's economic empowerment to improved health outcomes for both women and their families. Benefits include uptake of family planning, improved nutrition, and reduced maternal and child mortality (43). The social entrepreneurship model has been used successfully to create incentives for the uptake of health services and behavior change in the context of HIV (54, 55), syphilis (56), and malaria (57). Social entrepreneurship programs are uniquely positioned to empower women who are vulnerable to sexually transmitted infections such as HIV due to gender disparity in social structures and relationships, such as income inequality, violence, and educational opportunities (58, 59). For example, Haitian women who participated in a microfinance program were less likely to report partner infidelity and more likely to report condom use with their partners than those who did not participate in the program (60). A micro-grant intervention called the SHAZ! Project reported a significant improvement in economic security and decreased HIV risk factors such as transactional sex or gender-based violence among the adolescent female orphans in Zimbabwe (61).

As noted in the conceptual model, resources, agency, and achievements were interrelated and indivisible for the empowerment of the Hope Ladies in our study. The Hope Ladies reported that improved resources in the form of supplemental income improved their ability to participate in financial and household decisions. The recognition of the Hope Ladies as a community resource for reproductive health widened their social network and gave them more social capital. Increased knowledge, self-confidence, and expanded social network empowered the Hope Ladies to see and advocate for the women in their communities by speaking out against the unequal power relations with their spouses, disparate access to healthcare, and fear of stigma in being diagnosed with cervical cancer.

As we posit in our causal pathway, the relational and economic empowerment of the Hope Ladies may be necessary but not sufficient to produce the pre-conditions of financial and logistical means of buying and selling HPV self-sampling kits. For example, the empowerment of the Hope Ladies alone cannot protect their time against competing household priorities such as caring for another family member. Leveraging the social entrepreneurship structure by increasing their financial incentives or providing resources that could reduce conflicting household roles (e.g., childcare) would bolster their empowerment and protect the Hope Ladies' time to sell more kits which would modify the effects of the contextual moderators.

Our study has several limitations. Our sample size was small, and the number of respondents varied in some survey questions because the data collection took place during the week Peru closed its borders due to the COVID-19 pandemic. These factors may negatively impact the generalizability of our findings. Secondly, the interviews took place in the participants' homes, which may have biased their responses due to privacy concerns. Although the interviewers were fluent in Spanish and the Hope Project administrators were not present during the interview, the presence of other study personnel may have contributed to social desirability bias. Third, because some of the survey data were collected 1 year later virtually due to the country lockdown, this may have introduced more recall bias. However, because our sample size was small and the Hope Ladies have not been working in the communities since interviewing in accordance with the pandemic precautions, we took the opportunity to strive for data completion. Despite the limitations, we rigorously evaluated the financial and relational empowerment of the Hope Ladies, using multiple well-established conceptual frameworks and mixed methods.

In conclusion, the participants in the community-based HPV self-sampling social entrepreneurship experienced improved financial and relational empowerment in the program. More research is needed to test and demonstrate the association between the Hope Ladies' empowerment and cervical cancer screening uptake by community members to scale this intervention to a broader population.

## Data Availability Statement

The original contributions presented in the study are included in the article/Supplementary Materials, further inquiries can be directed to the corresponding author/s.

## Ethics Statement

The study was approved by the International Review Boards of Cayetano Heredia University (#103900), Duke University (#2020-0376), and University of Washington (STUDY00010676). All participants gave written consent in Spanish. All methods were carried out in accordance with relevant guidelines and regulations.

## Author Contributions

MS, PG, RB, and SG made substantial contributions to the conception and the design of the work. MD, MV, MC, and PG contributed to data acquisition. MS and MD conducted data analysis, interpretation of the data, and drafted the original manuscript. PG, NR, MK, KÁ, RB, SI, and SG provided substantial review and revision to the manuscript. All authors contributed to the article and approved the submitted version.

## Funding

This work was supported by the University of Washington: School of Nursing, the United States Agency for International Development (USAID-DIV 7200AA19FA00001), Duke Universities Bass Connections project titled Analysis of Bringing Elements of Referral Services to Community Care, and GMaP Region 5 program of the Fred Hutch/University of Washington Cancer Consortium 3P30CA015704-46S5.

## Conflict of Interest

The authors declare that the research was conducted in the absence of any commercial or financial relationships that could be construed as a potential conflict of interest.

## Publisher's Note

All claims expressed in this article are solely those of the authors and do not necessarily represent those of their affiliated organizations, or those of the publisher, the editors and the reviewers. Any product that may be evaluated in this article, or claim that may be made by its manufacturer, is not guaranteed or endorsed by the publisher.

## References

[1] García-Jurado A Pérez-Barea JJ Nova RJ. A new approach to social entrepreneurship: a systematic review and meta-analysis. Sustainability. (2021) 13:27545. 10.3390/su13052754

[2] Phillips W Lee H Ghobadian A O'Regan N James P. Social Innovation and Social Entrepreneurship: A Systematic Review. Group Organ Manag. (2014) 40:428–61. 10.1177/1059601114560063

[3] Short JC Moss TW Lumpkin GT. Research in social entrepreneurship: past contributions and future opportunities. Strateg Entrepreneurship J. (2009) 3:161–94. 10.1002/sej.69

[4] Bacq S Janssen F. The multiple faces of social entrepreneurship: A review of definitional issues based on geographical and thematic criteria. Entre Regl Dev. (2011) 23:373–403. 10.1080/08985626.2011.577242

[5] Sassmannshausen, SP, Volkmann, C,. A Bibliometric based Review on Social Entrepreneurship and its Establishment as a Field of Research, Schumpeter Discussion Papers, No. 2013-003, University of Wuppertal, Schumpeter School of Business and Economics, Wuppertal, Available online at: http://nbn-resolving.de/urn:nbn:de:hbz:468-20130423-111122-6

[6] Mosher-Williams R Association Association for Research on Nonprofit O Voluntary A. Research on Social Entrepreneurship: Understanding and Contributing to an Emerging Field. Washington, DC: Aspen Institute (2006). p. 7–150.

[7] Huybrechts B Nicholls A. Social Entrepreneurship: Definitions, Drivers and Challenges. Wiesbaden: Gabler Verlag (2012). p. 31-48. 10.1007/978-3-8349-7093-0_2

[8] Volkmann CK Tokarski KO Ernst K Volkmann CK Tokarski KO Ernst K. Social Entrepreneurship and Social Business: an Introduction and Discussion With Case Studies. Wiesbaden: Springer Gabler. (2012). 10.1007/978-3-8349-7093-0

[9] Gupta P Chauhan S Paul J Jaiswal MP. Social entrepreneurship research: A review and future research agenda. J Bus Res. (2020) 113:209–29. 10.1016/j.jbusres.2020.03.032

[10] Macke J Sarate JAR Domeneghini J. Silva KAd. Where do we go from now? Research framework for social entrepreneurship. J Clean Prod. (2018) 183:677–85. 10.1016/j.jclepro.2018.02.017

[11] Akbulaev N Aliyev Y Ahmadov T. Research models for financing social business: theory and practice. Heliyon. (2019) 5:e01599. 10.1016/j.heliyon.2019.e01599 31193392 PMC6527910

[12] Mair J Battilana J Cardenas J. Organizing for society: a typology of social entrepreneuring models. J Bus Ethics. (2012) 111:353–73. 10.1007/s10551-012-1414-3

[13] Mair J Martí I. Social entrepreneurship research: a source of explanation, prediction, and delight. J World Bus. (2006) 41:36–44. 10.1016/j.jwb.2005.09.002

[14] Marshall RS. Conceptualizing the international for-profit social entrepreneur. J Bus Ethics. (2011) 98:183–98. 10.1007/s10551-010-0545-7

[15] Zahra SA Gedajlovic E Neubaum DO Shulman JM A A typology of social entrepreneurs: motives search processes and ethical challenges. J Bus Ventur. (2009) 24:519–32. 10.1016/j.jbusvent.2008.04.007

[16] Campbell S. Social entrepreneurship: how to develop new social-purpose business ventures. Health Care Strateg Manage. (1998) 16:17–8. 10179045

[17] Murray RC-G J;Mulgan G. The Open Book of Social Innovation. The Young Foundation. London: National Endowment for Science, Technology and the Art, Young Foundation. (2010).

[18] Zadek S Thake S. Send in the social entrepreneurs. New Statesman. (1997) 20:31.

[19] Austin J Stevenson H. Wei–Skillern J. Social and Commercial Entrepreneurship: Same, Different, or Both? ENTREP THEORY PRACT. (2006) 30:1–22. 10.1111/j.1540-6520.2006.00107.x

[20] Chant S. Exploring the feminisation of poverty in relation to womeé s work and home-based enterprise in slums of the Global South. Int J Gend Entrep. (2014) 6:296–316. 10.1108/IJGE-09-2012-0035

[21] Lim YW Chia A. Social entrepreneurship: improving global health. Jama. (2016) 315:2393–4. 10.1001/jama.2016.4400 27299615

[22] Haugh HM Talwar A. linking social entrepreneurship and social change: the mediating role of empowerment. J Bus Ethics. (2016) 133:643–58. 10.1007/s10551-014-2449-4

[23] Kabeer N. Resources, agency, achievements: reflections on the measurement of women's empowerment. Dev Change. (1999) 30:435–64. 10.1111/1467-7660.00125

[24] Kabeer N. Economic pathways to women's empowerment and active citizenship: what does the evidence from Bangladesh tell us? J Dev Stud. (2017) 53:649–63. 10.1080/00220388.2016.1205730

[25] Datta PB Gailey R. Empowering women through social entrepreneurship: case study of a women's cooperative in India. ENTREP THEORY PRACT. (2012) 36:569–87. 10.1111/j.1540-6520.2012.00505.x

[26] Mair J Schoen O. Successful social entrepreneurial business models in the context of developing economies. Int J Emerg Mark. (2007) 2:54–68. 10.1108/17468800710718895

[27] Santos FM A A positive theory of social entrepreneurship. J Buss Ethics. (2012) 111:335–51. 10.1007/s10551-012-1413-4

[28] Bruni L AG Serrano B Mena M Gómez D Muñoz J Bosch FX de Sanjosé S. Human Papillomavirus and Related Diseases in Peru. Summary Report ICO/IARC Information Center on HPV and Cancer (HPV Information Center), (2019).

[29] Bendezu-Quispe G Soriano-Moreno AN Urrunaga-Pastor D Venegas-Rodríguez G Benites-Zapata VA. Asociación entre conocimientos acerca del cáncer de cuello uterino y realizarse una prueba de Papanicolaou en mujeres peruanas. Rev Peru Med Exp Salud Publica. (2020) 37:17–24. 10.17843/rpmesp.2020.371.4730 32520183

[30] Sankaranarayanan R Anorlu R Sangwa-Lugoma G Denny LA. Infrastructure requirements for human papillomavirus vaccination and cervical cancer screening in sub-Saharan Africa. Vaccine. (2013) 31 Suppl 5:F47–52. 10.1016/j.vaccine.2012.06.066 24331747

[31] Basu P Meheus F Chami Y Hariprasad R Zhao F Sankaranarayanan R. Management algorithms for cervical cancer screening and precancer treatment for resource-limited settings. Int J Gynaecol Obstet. (2017) 138 (Suppl 1):26–32. 10.1002/ijgo.12183 28691336

[32] Aguilar A Pinto JA Araujo J Fajardo W Bravo L Pinillos L. Control of cervical cancer in Peru: Current barriers and challenges for the future. Mol Clin Oncol. (2016) 5:241–5. 10.3892/mco.2016.926 27446557 PMC4950606

[33] Paz-Soldán VA Bayer AM Nussbaum L Cabrera L. Structural barriers to screening for and treatment of cervical cancer in Peru. Reprod Health Matters. (2012) 20:49–58. 10.1016/S0968-8080(12)40680-2 23245408 PMC3839786

[34] Stenning-Persivale K Franco MJS Cordero-Morales A Cruzado-Burga J Poquioma E Nava ED. The mortality-incidence ratio as an indicator of five-year cancer survival in metropolitan Lima. Ecancermedicalscience. (2018) 12:799. 10.3332/ecancer.2018.799 29456616 PMC5813917

[35] Organization WH. WHO Consolidated Guideline on Self-Care Interventions for Health: Sexual and Reproductive Health and Rights. Geneva: World Health Organization (2019). Available online at: https://apps.who.int/iris/bitstream/handle/10665/325480/9789241550550-eng.pdf?ua=1. 31334932

[36] Moran F Carcamo C Valderrama M Garcia PJ. [Preferences and satisfaction toward a screening program with self-administered human papilloma virus detection tests]. Rev Peru Med Exp Salud Publica. (2017) 34:228–32. 10.17843/rpmesp.2017.342.2453 29177380

[37] The Hope Project Peru. Hope: Proof of Concept (2019) [cited 2019 October 4]. Available online at: https://hopeperuproject.org/proof-of-concept/.

[38] Skrabut K. Housing the contingent life course: domestic aspiration and extreme poverty in Peruvian Shantytowns. City Soc. (2018) 30:263–88. 10.1111/ciso.12145

[39] Górska K. Todas las sangres - Peruvian multiculturalism in a migrant settlement of metropolitan Lima 1. Ad Americam. (2016) 17:79. 10.12797/AdAmericam.17.2016.17.06

[40] Alianza, Para El Progreso,. Plan de Gobierno Distrito de Ventanilla-Callao 2019-2022. Available online at: https://cde.3.elcomercio.pe/doc/0/1/7/1/3/1713441.pdf (accessed March 1, 2021).

[41] Plan nacional de prevención y control de cáncer de cuello uterino 2017–2021. (Resolución Ministerial N° 440-2017/MINSA)

[42] Shin MB Garcia PJ Saldarriaga EM Fiestas JL Ásbjörnsdóttir KH Iribarren SJ. Cost of community-based human papillomavirus self-sampling in Peru: a micro-costing study. Lancet. (2022) 8:100160. 10.1016/j.lana.2021.100160 35528707 PMC9075528

[43] Golla AM Malhotra A Nanda P Mehra R. Understanding Measuring Women's Economic Empowerment: Definition, Framework Indicators. International Center for Research on Women. (2018). Available online at: https://www.icrw.org/wp-content/uploads/2018/04/ICRW_MeasuringWomensEconomicEmpowerment_v4_WebReady.pdf (accessed March 1, 2021).

[44] Uhl-Bien M. Relational Leadership Theory: Exploring the Social Processes of Leadership and Organizing. Leadersh Q. (2006) 17:654–76. 10.1016/j.leaqua.2006.10.007

[45] Manuere F Phiri N A. Literature Review of Women Empowerment and Development in Zimbabwe: A look at New Insights and Perspectives. J Public Admin. (2018) 8:57. 10.5296/jpag.v8i4.13818

[46] Dekker M editor. Promoting Gender Equality and Female Empowerment: a Systematic Review of the Evidence on Property Rights, Labor Markets, Political Participation and Violence Against Women. Leiden: African Studies Center (2013). p. 126.

[47] O'Hara C Clement F. Power as agency: A critical reflection on the measurement of women's empowerment in the development sector. World Dev. (2018) 106:111–23. 10.1016/j.worlddev.2018.02.002

[48] Leder S. Linking Women's Empowerment and their Resilience. CGIAR Research Program on Water, Land and Ecosystems (WLE). (2016). 27p.

[49] Christens BD. Toward Relational Empowerment. Am J Community Psychol. (2012) 50:114–28. 10.1007/s10464-011-9483-5 22094588

[50] Creswell JW. A Concise Introduction to Mixed Methods Research. California: SAGE publications (2021).

[51] Elo S Kyngas H. The qualitative content analysis process. J Adv Nurs. (2008) 62:107–15. 10.1111/j.1365-2648.2007.04569.x 18352969

[52] Guetterman TC Fetters MD Creswell JW. Integrating quantitative and qualitative results in health science mixed methods research through joint displays. Ann Fam Med. (2015) 13:554–61. 10.1370/afm.1865 26553895 PMC4639381

[53] Lewis CC Klasnja P Powell BJ Lyon AR Tuzzio L Jones S. From classification to causality: advancing understanding of mechanisms of change in implementation science. Front Public Health. (2018) 6(136). 10.3389/fpubh.2018.00136 29868544 PMC5949843

[54] Sherer RD. Jr., Bronson JD, Teter CJ, Wykoff RF. Microeconomic loans and health education to families in impoverished communities: implications for the HIV pandemic. J Int Assoc Physicians AIDS Care. (2004) 3:110–4.s 10.1177/154510970400300402 15768731

[55] Viravaidya M Wolf RC Guest P. An assessment of the positive partnership project in Thailand: key considerations for scaling-up microcredit loans for HIV-positive and negative pairs in other settings. Glob Public Health. (2008) 3:115–36. 10.1080/17441690801903070 19288366

[56] Tucker JD Muessig KE Cui R Bien CH Lo EJ Lee R. Organizational characteristics of HIV/syphilis testing services for men who have sex with men in South China: a social entrepreneurship analysis and implications for creating sustainable service models. BMC Infect Dis. (2014) 14:601. 10.1186/s12879-014-0601-5 25422065 PMC4247875

[57] Allen LK Hetherington E Manyama M Hatfield JM van Marle G. Using the social entrepreneurship approach to generate innovative and sustainable malaria diagnosis interventions in Tanzania: a case study. Malar J. (2010) 9:42. 10.1186/1475-2875-9-42 20128922 PMC2827419

[58] Sumartojo E. Structural factors in HIV prevention: concepts, examples, and implications for research. AIDS. (2000) 14:S3–S10. 10.1097/00002030-200006001-00002 10981469

[59] Auerbach JD Parkhurst JO Cáceres CF. Addressing social drivers of HIV/AIDS for the long-term response: conceptual and methodological considerations. Glob Public Health. (2011) 6:S293–309. 10.1080/17441692.2011.594451 21745027

[60] Rosenberg MS Seavey BK Jules R Kershaw TS. The role of a microfinance program on HIV risk behavior among Haitian women. AIDS Behav. (2011) 15:911–8. 10.1007/s10461-010-9860-3 21153762

[61] Dunbar MS Kang Dufour M-S Lambdin B Mudekunye-Mahaka I Nhamo D Padian NS. The SHAZ! project: results from a pilot randomized trial of a structural intervention to prevent hiv among adolescent women in Zimbabwe. PLoS ONE. (2014) 9:e113621. 10.1371/journal.pone.0113621 25415455 PMC4240618

