# Supplementary material for: Evaluation of Women's Empowerment in a Community-Based Human Papillomavirus Self-Sampling Social Entrepreneurship Program (Hope Project) in Peru: A Mixed-Method Study
Source: Front Public Health. 2022 Jun 13;10:858552. doi: 10.3389/fpubh.2022.858552 (PMC9236182; doi:10.3389/fpubh.2022.858552)
Supplement: Supplementary file 1 [file Data_Sheet_1.docx]

Supplementary Material

# Relational and financial empowerment survey

ENGLISH TRANSLATION

**The survey will take at most 10 minutes and will be given orally.**

***Research Question(s):***

- Does the implementation of a combination of a microfinancing and peer-education program called the **Hope model** increase relational empowerment and financial autonomy within the household for the peer-educators and the women they serve in Ventanilla, Peru?

***To understand how the Hope initiative impacts the Hope ladies’ relational empowerment***

1. Insight to women’s impact of the Hope micro-business services in promoting women’s social awareness and their capacity to influence social norms and practices
   1. Would you say that your number of social contacts in and outside of the family has increased since the beginning of your Hope Lady journey?
      1. Can respond on a 4-point scale: “strongly disagree” (0), “ disagree” (1), “ agree” (2), “strongly agree” (3).
   2. Would you say that you have been able to help other women in times of need since the beginning of your Hope Lady journey?
      1. Can respond on a 4-point scale: “strongly disagree” (0), “ disagree” (1), “ agree” (2), “strongly agree” (3).
   3. Would you say that you have been able to increase your visits to a healthcare provider unaccompanied to satisfy personal needs since the beginning of your Hope Lady journey?
      1. Can respond on a 4-point scale: “strongly disagree” (0), “ disagree” (1), “ agree” (2), “strongly agree” (3).
   4. Would you say you have felt confident due to learning about your reproductive health and how to prevent certain diseases compared to the beginning of your Hope Lady journey?
      1. Can respond on a 4-point scale: “strongly disagree” (0), “ disagree” (1), “ agree” (2), “strongly agree” (3)

***To understand how the Hope initiative impacts financial autonomy for the Hope ladies***

1. Assessing Financial independence based on autonomy/influence from three questions, based on the past and present, representing a Hope Lady’s autonomy and influence in purchases.
   1. In the present, can you decide how to spend any household monetary earnings?
      1. Can respond on a 4-point scale: “often” (3), “sometimes” (2), “rarely” (1), or “never” (0).
   2. In the past, could you decide how to spend any household monetary earnings?
      1. Can respond on a 4-point scale: “often” (3), “sometimes” (2), “rarely” (1), or “never” (0).
   3. In the present, do you have a say in the purchase of large household assets?
      1. Can respond on a 4-point scale: “often” (3), “sometimes” (2), “rarely” (1), or “never” (0).
   4. In the past, could you have a say in the purchase of large household assets?
      1. Can respond on a 4-point scale: “often” (3), “sometimes” (2), “rarely” (1), or “never” (0).

SPANISH TRANSLATION

**Encuesta de relaciona**l y **financiera empoderamiento**

***La encuesta tomará como máximo 10 minutos y el contenido se dará oralmente.***

***Pregunta(s) de investigación:***

- ¿La implementación de una combinación de un programa de microfinanciación y educación entre pares llamado modelo Hope aumenta el empoderamiento relacional y la autonomía financiera dentro del hogar para las educadoras pares y las mujeres a las que sirven en Ventanilla, Perú?

***Para entender cómo la iniciativa Hope impacta el empoderamiento relacional de las Hope Ladies***

1. Conocimiento del impacto de las mujeres de los servicios de microempresas Hope en la promoción de la conciencia social de las mujeres y su capacidad para influir en las normas y prácticas sociales.
   1. ¿Diría que su número de contactos sociales dentro y fuera de la familia ha aumentado desde el comienzo de su viaje Hope Lady?
      1. Puede responder en una escala de 4 puntos: "totalmente en desacuerdo" (0), "en desacuerdo" (1), "de acuerdo" (2), "muy de acuerdo" (3).
   2. ¿Diría que ha podido ayudar a otras mujeres en momentos de necesidad desde el comienzo de su viaje Hope Lady?
      1. Puede responder en una escala de 4 puntos: "totalmente en desacuerdo" (0), "en desacuerdo" (1), "de acuerdo" (2), "muy de acuerdo" (3).
   3. ¿Diría que ha podido aumentar sus visitas a un proveedor de atención médica no acompañado para satisfacer sus necesidades personales desde el comienzo de su trabajo Hope Lady?
      1. Puede responder en una escala de 4 puntos: "totalmente en desacuerdo" (0), "en desacuerdo" (1), "de acuerdo" (2), "muy de acuerdo" (3).
   4. ¿Diría que se ha sentido confiado debido a aprender sobre su salud reproductiva y cómo prevenir ciertas enfermedades en comparación con el comienzo de su trabajo Hope Lady?
      1. Puede responder en una escala de 4 puntos: "totalmente en desacuerdo" (0), "en desacuerdo" (1), "de acuerdo" (2), "muy de acuerdo" (3).

***Comprender cómo la iniciativa Hope impacta la autonomía financiera de las Hope Ladies***

2. Evaluar la independencia financiera basada en la autonomía / influencia de tres preguntas, basadas en el pasado y el presente, que representan la autonomía e influencia de Hope Lady en las compras.

- 1. En el presente, ¿puedes decidir cómo gastar los ingresos monetarios de su hogar?
     1. Puede responder en una escala de 4 puntos: "a menudo" (3), "a veces" (2), "rara vez" (1) o "nunca" (0).
  2. En el pasado, ¿podría decidir cómo gastar los ingresos monetarios de su hogar?
     1. Puede responder en una escala de 4 puntos: "a menudo" (3), "a veces" (2), "rara vez" (1) o "nunca" (0).
  3. En el presente, ¿a usted se le permite opinar sobre la compra de grandes activos domésticos?
     1. Puede responder en una escala de 4 puntos: "a menudo" (3), "a veces" (2), "rara vez" (1) o "nunca" (0).
  4. En el pasado, ¿a usted se le permitía opinar sobre la compra de grandes activos domésticos?
     1. Puede responder en una escala de 4 puntos: "a menudo" (3), "a veces" (2), "rara vez" (1) o "nunca" (0).

# Qualitative interview guide questions

1. Are you currently active as a Hope lady?
2. Please walk us through what a typical day looks like for your Hope Lady work.
3. How many women do you visit and test each day?
4. How many times do you have to interact with a woman in order for them to buy and take an HPV test?
5. Do you believe that the Hope program is vital to your community? If so, why or why not?
6. How much time have you been working as a Hope lady?
7. Why do you choose to educate your fellow female peers? (**RLT**)
8. Women's empowerment is defined as the process by which women acquire greater control over their own lives, the circumstances surrounding it, and the elements that are part of it, such as reproductive health.
   1. How has this program empowered you? In other words, how has it affected your access to health care and female education? **(ICRW, RLT**)
9. How does the Hope micro-business model help empower you in your everyday life or improved your earning opportunities? **(ICWR)**
10. What new skills have you learned since becoming a Hope lady, those being related to education, finances, and social? **(ICWR, RLT)**
11. What do you teach women when you interact with them and teach them about women’s health? (**RLT**)
12. Would you describe your ability to balance your Hope Lady duties with your personal life as: 1) very easy, 2) easy, 3) difficult 4) very difficult **(RLT)**
13. Have you faced any setbacks that have kept you from your Hope Lady work? If so, what are they, and what brings your back to doing the Hope Lady work?
14. What would help you maintain your Hope Lady position?

# Cross-Case Comparison and Mixed Methods Integration of Interviews and Survey Results

| **General category** | **Sub-category/ Pre-determined codes** | **Corresponding survey question** | **Themes, reference counts, and demonstrative quotes** | **Corresponding survey response /Mixed-method integration** | **Results interpretation** |
| --- | --- | --- | --- | --- | --- |
| Resources | Individual  Gender norms and roles within the household  Individual, within household relationships as a context | RLT 1.a. “Would you say that your number of social contacts within and outside the family has increased since the beginning of your HOPE lady journey?”  RLT 1.b. “Would you say that you have been able to help other women in moments of need since the beginning of your HOPE lady journey?” | ***Theme: Role in the household sometimes conflicting with the role as a Hope Lady (n= 15, References –35)*** | | Divergence: 5 of the 5 participants agreed/totally agreed that their social contacts increased and were able to help community women. 1 of 5 participants are active.  Interviews revealed all of them feel overwhelmed at times due to conflicting household roles, unable to meaningfully engage in increased social contacts and sometimes feeling guilty for pausing their Hope Project activities. |
|  |  |  | P17: I have a baby. When she grows a little more, I don't think I will have any obstacles in the Hope Project. (age 35) | P17 answered 3 (totally agree) on 1.b. and 3 (totally agree) on 1.a. |  |
|  |  |  | P3: I have my mother-in-law in my care. She needs me to take care of her...because she cannot get out of bed. I go [out to sell the kits], but with the thought, 'what if she suddenly falls out of bed,' or I do not know she will urinate on herself. With that thought I go, sometimes I say, ‘I continue or I do not continue,’ and sometimes I stop. My friends tell me, ‘Don't stop, keep going for us.’ (age 33) | P3 answered 3 (totally agree) on both survey questions. |  |
|  |  |  | P2: I have to take my son to school, I have to cook and sometimes because of those issues they [the women who screened positive] don't go to the hospital [for triage]. (age 45) | P2 answered 3 (totally agree) on question 1.a., and 2 (agree) on question 1.b. |  |
|  |  |  | P5: Unfortunately, my mother passed away and I have stayed with my father…I do not have that time to reestablish myself with the Hope Project. My father lives far away and my father is an eighty-three-year-old person. That is the impediment I have, my dad doesn't want to come here! So, I have to go to his house to see him. (age 33) | P5 answered 3 (totally agree) on both questions. |  |
|  |  |  | P8: Unfortunately, I have not had enough time, because how I tell the truth, sometimes I am left alone with the store, the house, the kitchen, the baby, the homework, and the school. I have lacked time, but if I had dedicated the time to it, the way one works, I bet I would have achieved more goals…the truth is, I have five children, and so many responsibilities that the time doesn't pay off. (age 44) | P8 answered 3 (totally agree) on both questions. |  |
|  | Community  Relationships as a collective identity (e.g., Hope Ladies) as a context | RLT 1.b  “Would you say that you have been able to help other women in moments of need since the beginning of your HOPE lady journey?” | ***Theme: Recognition from the community as a resource for women***  ***(n= 10, References – 12)*** | | Convergence: The client statements from the qualitative interviews show that the community values the Hope ladies and recognizes them as leaders is supported by the quantitative results showing that 3 out of 3 quoted participants agree that they have been able to help other women since starting active work with the Hope program. |
|  |  |  | P2: They [the community women] talk to me more because you know in the hospital they will hardly talk to them like we [Hope Ladies] talk to them. They are always be afraid to ask the doctor, or that [the doctors] will ask if they have not understood. We as leaders of our community know our neighbors…They comment on the program [Hope Project] and they look for us and they call us about this topic. (age 45) | P2 answered 2 (agree) to RLT question 1.b.  The transcript showed that this participant greatly valued being able to help her community, specifically in being more relatable and less scary than a medical doctor. |  |
|  |  |  | P3: Sometimes, my difficulties are the lack of communication, sometimes the ladies doubt, when you go you have not heard anywhere, they say: but, it will be true or it will be false because, well, it is 10 soles or how much, sometimes They come to fool us, sometimes, no! no! no! Suddenly, it's a lie, they close the door! Sometimes it is discouraging when you go somewhere they have not heard of us before, and they close the door on us. But now there is a lot of communication, [Hope Project] has been on TV, they have been on the radio. The community has listened, so when we knock on the door we say: we are from Hope! They say, “Oh that program! I was waiting at my house for a knock on my door!” (age 33) | P3 answered 3 (totally agree) to the RLT question 1.b. |  |
|  |  |  | P13: Sometimes, the women [in the community] think we are doctors and nurses and ask us about women’s illnesses... so we correct them and find out who they should go to. (age 47) | P13 answered 3 (totally agree) to RLT question 1.b.  The transcript showed that this participant has gone above and beyond her commitments with Hope by assisting community members with navigating the healthcare system for other illnesses, even supporting women through difficult personal situations. |  |
|  | Community  Relationships as a collective identity (e.g., Hope Ladies) as a context | N/A | ***Theme: The camaraderie with other Hope Ladies (n= 9, References – 20)*** | | Half (n=9, 45%) of the Hope Ladies who were interviewed commented on enjoying the peer support with other Hope Ladies. |
|  |  |  | P10: We would agree with other colleagues [Hope Ladies]…and we would go out in a group, because it is less tedious [than] when you are alone. (age 64) | |  |
|  |  |  | P19: We are working with Cayetano throughout this project, so that [the Hope Project] grows and we can amplify the good work. (age 54) | |  |
| Agency | Individual  Self-efficacy | RLT 1.c. “Would you say that you have been able to increase your unaccompanied visits to a health care provider to meet your personal needs since the beginning of your job HOPE lady?” | ***Theme: Increased knowledge (n= 20, References – 27)*** | | Convergence: The quoted participants discussed how they experienced improved ability to speak with other women because of the increased knowledge about the reproductive health system.  P7 and P16 also discussed how they accompanied their clients (those who tested positive for HPV) to the follow-up visit upon their request. They said that many HPV positive women do not want to go to the clinic accompanied for visual inspection due to fear of cancer diagnosis, encountering a male provider, and long wait time. After becoming a Hope Lady, they feel that they can be an advocate for these women. |
|  |  |  | P7: Well, with hope, I have lost the shame of communicating with people, because before I was not capable, when I started I was very shy, but now I have enough skills. I have acquired that with [Hope Project] because of the training that they also give us, they support us in everything that we do, we also consult with them. (age 46) | P7 responded 3 (totally agree) for 1.c. |  |
|  |  |  | P9: I have learned a lot and of which, well, it is quite beneficial, because that way I am educating myself, that is, I am learning and I am really glad that these programs exist because they educate you, you learn from this, so it's very good! I really congratulate the program, because if it weren't for this, I wouldn't even have taken the test. (age 44) | P9 responded 1 (disagree) for 1.c. |  |
|  |  |  | P16: There were two ladies in particular [from the 2015 pilot] that we saved her from cervical cancer. One had her uterus removed. She thanked me, she wanted to pay me, so, I said no. You feel good because you say, ‘well I did something good and look here is the result.’ I felt very good. (age 47) | P16 responded 3 (totally agree) for 1.c and 3 (totally agree). |  |
|  | Individual  Self-confidence | RLT 1.d.  “Would you say that you have felt confident because of learning about your reproductive health and how to prevent certain diseases compared to starting your job HOPE lady?” | ***Theme: Confidence to speak about reproductive health***  ***Improved ability to communicate and express themselves (n= 12, References – 19)*** | | Convergence: 4 out of 4 quoted participants answered that they ‘totally agree’ when asked if they felt more confident because of learning about their reproductive health since joining Hope. This supports the qualitative results where over half of the women directly stated that participation as a Hope lady had improved their confidence. Despite some women feeling nervous to start the program, all grew into their roles as Hope Ladies. |
|  |  |  | P11: Well, in my case, it has helped me to have more confidence in words, that is, in being able to express myself with confidence what I am talking about. (age 33) | P11 answered 3 (totally agree) to RLT survey question 1.d.  In the transcript this participant mentioned several times that the Hope project had improved her confidence and public speaking skills. |  |
|  |  |  | P3: [Hope Project] has also helped me to express myself, because before I did not speak...But, this program has helped me connecting with other people, more than economically. (age 33) | P3 answered 3 (totally agree) to RLT survey question 1.d.  The transcript showed that this participant has grown a lot during her time with Hope and obtained skills, such as social media. |  |
|  |  |  | P13: [The Hope Project] has taught me to talk to women and lose my shame. We [the Hope Ladies] lose our fear with the talks and training. Hope’s support has been very important to us because we have educated ourselves through it. (age 47) | P13 answered 3 (totally agree) to RLT survey question 1.d. |  |
|  |  |  | P16: The first thing is to learn to know and love ourselves [to be take the test]. Above all the love of oneself. (age 47) | P16 answered 3 (totally agree) to RLT survey question 1.d. |  |
|  | Community  Change in relational dynamics (new approaches, values, attitudes, behaviors, and ideologies) (RLT) | N/A | ***Theme: Ability to speak against machismo culture and advocate for women to make autonomous decisions (n= 18, References – 27)*** | | Almost all (n=18, 90%) of the Hope Ladies who were interviewed mentioned male-dominant culture (machismo) in the households as a barrier to selling the HPV self-sampling kits. Three (15%) Hope Ladies reported clients who buy the HPV self-sampling kits in secret, without informing their husbands. A quarter of all study participants (n=5, 25%) mentioned their clients shared that they sometimes experience it. |
|  |  |  | P4: Yes, it is an important factor, for the husbands to give them permission. Many times they say, "No, my husband does not want to" and they have to talk with the husband. That is, more than anything, machismo. (age 50) | |  |
|  |  |  | P10: Women are well trodden, sometimes it depends a lot… I say to them, “Who is the one who is going to show their body [to the doctor]? Who is the one who is going to get sick? Your husband or you?” …There are cultural barriers…because the liberation of women is also being imposed! (age 64) | |  |
|  |  |  | P5: We are not talking about the test anymore; we talked and encountered different problems. We are like a heart doctor, they already believe us [heart] doctors, because they ask us different things. We try to do that, to be able to lift the woman, so that they are not left saying ‘Oh, I'm going to ask my husband's permission!’ (age 33) | |  |
|  |  |  | P6: The empowerment that [the Hope Project] brings to us, that other institutions cannot, is women’s self-realization, their power to decide themselves, not to ask their partner. (age 48)  P6: I discovered how much violence [there is in the homes], already. (age 48) | |  |
|  |  |  | P7: Machismo. Of the men who ... I have had quite a few cases in which they have not been allowed to take the test or the woman has to ask for permission. I tell her, "You don't have to ask for permission! Because he is not your dad, he is your partner, he is your husband...You are your body." That is machismo. (age 46) | |  |
|  |  |  | We always try to solve the doubts that people have, and also empower women, because sometimes some women do not want to do it, because first they have to consult with the husband first, then what we do it is to say to her, "She is the own owner of her body, of her health! There are men who do not want to let them get tested. But if [you have HPV] it is your body that will suffer the consequences." We help them, so that they can become aware that the decision is in oneself, and that we do not depend on anyone. We say, "We have come alone and we are going to leave alone, so each one is the owner of what to do and what decisions to make." And that is what I have learned with Hope Project. | |  |
|  |  |  | P16: Many women say, ‘Oh, I’m going to tell my husband!’ and the husbands say ‘No, how are you going to do such nonsense test!’ I say to the women, ‘why do you have to consult them? You are the owner of your person, you are a woman, health is yours not his…you have to take care of yourself.’ (age 47) | |  |
|  |  |  | P2: Mostly women do it [HPV self-sampling] secretly (age 45) | |  |
|  |  |  | P3: As I said, of 100%, there is always 1%, husbands who do not want [HPV self-sampling]. But they [wives of husbands who do not want HPV self-sampling] do it in secret. Many women do it in secret [without telling their husbands]. (age 33) | |  |
|  |  |  | P16: [some women say] “I’m going to pay you secretly, come on a day my husband is not here.” (age 47) | |  |
|  |  |  | P6: I discovered how much violence [there is in the homes], already. (age 48) | |  |
|  |  |  | P20: No, they [the spouses] don’t let women get tested [for HPV]. That’s where I’ve discovered how much violence there is. [The men] made me work harder, thinking about what I should do to meet with the women. (age 51) | |  |
|  |  |  | P11: Some women don’t do [the HPV self-sampling] out of fear of [their spouses]. They say, ‘my husband will ask me why I’m taking the test! He might think it’s because I doubt [his fidelity].’ (age 33) | |  |
| Achievement | Individual  Control over assets | ICRW 2.a.  “Currently do you decide how to spend money in your household?”  ICRW 2.b.  “In the past before joining HOPE project, did you decide how to spend money in your household?” | ***Theme: Increased economic assets (n= 13, References – 24)*** | | Convergence: The three quoted participants had a varying level of change in how often they made household spending decisions before participating in the Hope Project and currently. All answered that they make spending decisions “often” currently (after participating in the Hope Project) and that their role as a Hope Lady helps them financially. |
|  |  |  | P2: Of course, it has helped me a lot, because, sometimes, here my husband does not earn more, weekly, sometimes, it helps me for my children's bus fares, which is daily for school. My husband does not bring daily, he is paid weekly. And you have to wait that week, while I go do this work. (age 45) | P2 answered 3 (often) to ICRW survey question 2.a.; and 2 (sometimes) to 2.b. |  |
|  |  |  | P3: It has helped me a lot, because in my free time I earn something else, to support my household, to help my husband with something. (age 33) | P3 answered 3 (often) to ICRW survey question 2.a.; and 1 (rarely) to 2.b. |  |
|  |  |  | P20: Yes, it helps [financially]. It is a job that helps you financially and that you are also helping other people, other women, I think it does help you financially. (age 51) | P20 answered 3 (often) to ICRW survey question 2.a.; and 0 (never) to 2.b. |  |
|  | Individual  Financial autonomy | ICRW 2.c.  “Currently, are you allowed to comment on the purchase of large domestic assets in the household?”  ICRW 2.d.  “In the past, were you allowed to comment on the purchase of large domestic assets in the household?” | ***Theme: Improved ability to make financial decisions (n= 8, References – 17)*** | | Divergence: All three quoted participants’ perception of their ability to make large domestic assets did not change before participating in the Hope Project and currently. |
|  |  |  | P16: I'm the one who works. I am a mother and father, I have a daughter and I am the one who says how much money comes into my house and how much I am going to spend. I try to balance what is my priority. (age 47) | P16 answered 3 (always) on 2.c. and 3 (always) on 2.d. |  |
|  |  |  | P5: I have always managed the house expenses, although I don't [work], I have always tried to solve all the house expenses. [My husband] is the one who contributes. (age 33) | P5 answered 3 (always) on 2.c. and 3 (always) on 2.d. |  |
|  |  |  | P20: Of course, the income [from the Hope Project] helps me! (age 51) | P20 answered 2 (sometimes) on 2.c. and 2 (sometimes) on 2.d. |  |
|  | Community  Relationship as an outcome | RLT 1.a  “Would you say that your number of social contacts within and outside the family has increased since the beginning of your HOPE lady journey?” | ***Theme: Widened social network***  ***Technology skills development (n= 11, References – 18)*** | | Convergence: In the qualitative data, the widened social network was often discussed in the context of social media and technology skills development. In the quantitative data, which was limited to the increased number of social contacts, all of the quoted participants agreed that the number of social contacts within and outside the family has increased since working as a Hope Lady.  It is clear from both the survey and the interview transcripts that the combination of technological skills and the widened social network gained increased the confidence the Hope Ladies have in their ability to learn and engage in new technologies such as using a smartphone. |
|  |  |  | P2: Now they call us, they leave our numbers, and other people who have never met call us, and you get to know more people. (age 45) | P2 answered 3 (totally agree) on 1.a. |  |
|  |  |  | P5: Social networks...the cell phone for me was nothing more like the phone that you go and answer, nothing at all! now I know, well, I chat everything. (age 33) | P5 answered 3 (totally agree) on 1.a. |  |
|  |  |  | P7: Yes, I have also learned a lot with social networks, because before joining [Hope Project], I did not even know I did not have a touch phone, I had a [phone with buttons], which was only to receive calls and messages…when I joined Hope Project, [a smartphone] was practically indispensable, well, the telephone and [the administrators] themselves have taught me to use it, they have taught me to enter the page, to enter the data. I have also learned everything about technology with the Hope Project, because before I didn't even care to pick up a phone, but now I do. (age 46) | P7 answered 3 (totally agree) on 1.a. |  |
|  |  |  | P18: Social networking apps, even though I already used them, it helped me to handle much more than before. Now we are in the time where everything is handled by the computer. (age 41) | P18 answered 3 (totally agree) on 1.a. |  |
|  |  |  | P13: I learned new skills to buy things online, enter the data into the system, to send it to the laboratory, and to write by WhatsApp…I have learned to communicate with people [using apps]. (age 47) | P13 answered 3 (totally agree) on 1.a. |  |
